# Effect of *Boswellia serrata* Resin Supplementation on Basic Chemical and Mineral Element Composition in the Muscles and Liver of Broiler Chickens

**DOI:** 10.1007/s12011-017-0966-6

**Published:** 2017-02-16

**Authors:** A. R. M. Al-Yasiry, B. Kiczorowska, W. Samolińska

**Affiliations:** 1 Department of Bromatology and Food Physiology, Institute of Animal Nutrition and Bromatology, University of Life Science, Akademicka Street 13, 20-950 Lublin, Poland; 2 Department of Animal Resources, University of Wasit, Al Kut, Wasit Iraq

**Keywords:** Broiler chicken, Meat, Liver, Mineral elements, Nutrients, *Boswellia serrata*

## Abstract

Supplementation of broiler chicken diets with resin rich in bioactive components, such as different boswellic acids, could improve productivity, chemical composition, and nutritive value of produced meat. The aim of the study was to assess the effect of different levels of *Boswellia serrata* (BSR) supplementation in broiler chicken diet on the basic chemical composition and the Ca, P, Mg, Fe, Zn, and Cu contents in the breast and drumstick muscles and liver. The analyses involved 200 Ross 308 chickens. The broiler chickens were fed with diets containing 0 (BSR0), 1.5 (BSR1.5), 2 (BSR2), and 2.5% (BSR2.5) of *B*. *serrata* resin. The supplementation of broiler chicken diets with 2.5% (BSR2.5) decreased linearly the ether extract in breast and drumstick muscles and the calorific value in drumstick muscles (*P* < 0.05). An increased level of Ca in the breast and drumstick muscles (control vs. BSR diets, linear, *P* < 0.05) and in the liver (control vs. BSR diets, quadratic, *P* < 0.05) as well as Mg in the drumstick muscles and liver (control vs. BSR diets, linear, *P* < 0.05) was noted in the BSR2 and BSR2.5 chicken groups. The BSR supplementation reduced Cu (in the breast and drumstick muscles and liver) (*P* < 0.05) and Zn retention (in the drumstick muscles) (C vs. BSR, linear, *P* < 0.05). *B*. *serrata* resin can be considered a good feed additive with a positive impact on the dietary value of poultry meat.

## Introduction

Currently, consumers seek safe, high-quality food. They attach greater importance to the production procedures, which in animal production, including poultry industry, are associated with the quality of animal nutrition. A group of food additives that is safe to consumers’ health and has a beneficial effect on the palatability and nutritional value of meat is phytobiotics [1]. The most popular plant supplements of feed mixtures include herbs and essential oils whose beneficial impact on the effectiveness of poultry production has already been comprehensively described in literature. The beneficial effect of phytobiotics in poultry nutrition is visible not only in improved health status but also in better production performance, e.g., higher weight gains, higher feed conversion rates, and improved nutritional and dietary values of meat. Additionally, phytobiotics increase the bioavailability of some micro- and macroelements, which are accumulated in tissues more easily. For instance, increased contents of Mg, Zn, and Cu were detected in poultry breast and thigh meat and in the liver [2–5]. Still, the poultry industry is seeking new nutrition solutions that can ensure a high level of production, which is inextricably associated with maintenance of a good health status of chickens throughout the feeding period. New phytobiotics, such as aromatic resins obtained from, e.g., trees of the genus *Boswellia* in the family *Burseraceae*, have appeared on the European market. So far, they have been applied in the traditional Ayurvedic medicine due to their anti-inflammatory, antiseptic, analgesic, antibacterial, anticancer, hepatoprotective, hypolipidemic, hypocholesterolemic, immunomodulatory, and antiproliferative action [6]. Such a broad spectrum of therapeutic properties of *Boswellia serrata* resin is ensured by the bioactive compounds contained therein. These include terpenes, with 11-keto-ß-acetyl-beta-boswellic, acetyl-11-keto-ß- boswellic, and acetyl-α- boswellic acids characterized by the highest biological activity [7].

Although *B*. *serrata* resin jest a botanical feed additive approved for use in poultry production according to the European Union Register of Feed Additives pursuant to Regulation (EC) No 1831/2003 [8], its potential application in poultry production is investigated by few researchers. Their studies indicate beneficial effects of *B*. *serrata* resin supplementation of broiler chicken diets on the production performance of these birds. Improvement of the poultry breeding performance, i.e., better weight gains, higher feed conversion rates, and even carcass slaughter performance, is associated with improved digestibility of nutrients contained in the diet. This results from microbiological stabilization of the gastrointestinal tract by the activity of boswellic acids. It results in optimization of the processes of digestion and absorption in the small intestine [9–11]. Greater bioavailability of nutrients and minerals may have a significant impact on the chemical composition of tissues. Supplementation of broiler chicken diets with resin rich in bioactive components, such as different boswellic acids, could improve productivity as well as the chemical composition and nutritive value of produced meat. From the consumer’s point of view, the greatest attention is focused on the nutrient content in tissues that are relevant for consumption, e.g., breast and thigh muscles or offal. The most popular element of offal is the liver, which is widely used in cookery. Therefore, the aim of the study was to determine the effect of different levels of *B*. *serrata* resin supplementation in diets for broiler chickens on the basic nutrients (dry matter, crude protein, ether extract, and crude ash), energy, and chosen mineral (Ca, P, Mg, Fe, Zn, and Cu) content in the breast and drumstick muscles and liver.

## Materials and Methods

### Birds, Diets, and Experimental Design

Two hundred 1-day-old broiler chickens (Ross 308, Aviagen, Cracow, Malopolskie Province, Poland) were randomly assigned to four dietary treatments with five cages per treatment and five females and five males per cage. The experiment lasted 6 weeks. The basal feed diets were made from cereal meal middlings (wheat and corn) and post-extraction soybean meal as recommended [12]. The broiler chickens were fed with three types of diets: starter (0 to 21 days), grower (22 to 35 days), and finisher (36 to 42 days); the detailed composition of the diets in each stage of animal feeding is presented in Table 1. The starter diet was fed to the broiler chickens in a crumbled form, and the grower and finisher diets in a granulated form. The resin was obtained from *B*. *serrata* trees by incision of a barkless trunk and left to dry in natural conditions (direct information from the seller). Fragmented natural *B*. *serrata* resin (BSR) was obtained commercially (Baghdad, Iraq). The chemical composition of the resin comprised 95.34% dry matter, ash 1.59% dry matter, protein 2.65% dry matter, fat 63.88% dry matter, and 2.38% gum resin acetyl-11-keto-ß-boswellic acid [9]. Dietary treatments consisted of the control (C) and the control supplemented with 1.5 (BSR1.5), 2 (BSR2), or 2.5% (BSR2.5) of *B*. *serrata* resin. All the diets were iso-energetic and iso-nitrogenous.

**Table 1:** Dietary ingredients and the nutrient content of the experimental diets (as-fed basis)

Item	Diets^a^											
	Starter (1–21 days)				Grower (22–35 days)				Finisher (36–42 days)			
	C	BSR1.5	BSR2	BSR2.5	C	BSR1.5	BSR2	BSR2.5	C	BSR1.5	BSR2	BSR2.5
Ingredients (%)												
Maize	30.0	30.0	30.0	30.0	29.0	29.0	29.0	29.0	30.0	30.0	30.0	30.0
Wheat	20.0	20.0	20.0	20.0	23.0	23.0	23.0	23.0	26.0	26.0	26.0	26.0
Soybean meal (46% crude protein)	39.47	39.47	38.97	38.47	36.76	37.26	36.76	36.26	32.13	32.13	31.63	31.13
*Boswellia serrata* resin	–	1.5	2.0	2.5	–	1.5	2.0	2.5	–	1.5	2.0	2.5
Soybean oil	6.0	4.5	4.5	4.5	7.0	5.0	5.0	5.0	8	6.5	6.5	6.5
Dicalcium phosphate	1.8	1.8	1.8	1.8	1.8	1.8	1.8	1.8	1.8	1.8	1.8	1.8
Limestone	1.2	1.2	1.2	1.2	1.0	1.0	1.0	1.0	0.7	0.7	0.7	0.7
NaCl	0.33	0.33	0.33	0.33	0.5	0.5	0.5	0.5	0.5	0.5	0.5	0.5
dl-methionine^b^	0.36	0.36	0.36	0.36	0.33	0.33	0.33	0.33	0.33	0.33	0.33	0.33
l-lysine^c^	0.34	0.34	0.34	0.34	0.36	0.36	0.36	0.36	0.34	0.34	0.34	0.34
Vitamin-mineral premix^d^	0.5	0.5	0.5	0.5	0.25	0.25	0.25	0.25	0.2	0.2	0.2	0.2
Chemical composition (g/kg)												
ME_n_^e^ (MJ/kg)	12.55	12.43	12.43	12.44	12.97	12.90	12.93	12.95	13.39	13.33	13.36	13.37
Crude protein	212.0	211.0	212.0	212.0	192.0	192.0	193.0	193.0	185.0	185.0	185.0	185.0
Lys	13.8	13.8	13.8	13.8	12.9	12.9	12.9	12.9	11.3	11.3	11.3	11.3
Met + cys	10.5	10.5	10.5	10.5	9.8	9.8	9.8	9.8	9.0	9.0	9.0	9.0
Na	1.7	1.7	1.7	1.7	1.7	1.7	1.7	1.7	1.7	1.7	1.7	1.7
Total calcium^f^	9.77	9.77	9.76	9.74	8.97	8.99	8.97	8.95	7.75	7.75	7.73	7.72
Total phosphorus^f^	7.31	7.31	7.29	7.26	7.24	7.27	7.24	7.22	7.14	7.14	7.11	7.08
Total magnesium^f^	1.85	1.85	1.84	1.83	1.79	1.80	1.79	1.77	1.69	1.69	1.68	1.67
Total iron^f^ (mg/kg)	424.34	424.34	423.41	422.48	413.35	414.28	413.35	412.42	395.7	395.7	394.77	393.84
Total zinc^f^ (mg/kg)	130.86	130.86	130.61	130.36	130.01	130.26	130.01	129.76	128.48	128.48	128.23	127.98
Total copper^f^ (mg/kg)	18.05	18.05	17.97	17.89	17.65	17.73	17.65	17.57	17.00	17.00	16.92	16.84

^a^Treatments: C = control diet without *Boswellia serrata* resin (BSR) supplementation; BSR1.5 = diet with 1.5% BSR supplementation; BSR2 = diet with 2.0% BSR supplementation; BSR2.5 = diet with 2.5% BSR supplementation

^b^Evonik Degussa Gmbh, Essen, Germany (per kilogram of 990 g met)

^c^Ajinomoto Eurolysine S.A.S., Amiens, France (per kilogram of 780 g lys)

^d^Added minerals and vitamins per kilogram of starter diet: Mn, 100 mg; I, 1 mg; Fe, 40 mg; Zn, 100 mg; Se, 0.15 mg; Cu, 10 mg; vitamin A, 15,000 IU; vitamin D_3_, 5000 UI; vitamin E, 75 mg; vitamin K_3_, 4 mg; vitamin B_1_, 3 mg; vitamin B_2_, 8 mg; vitamin B_6_, 5 mg; vitamin B12, 0.016 mg; biotin, 0.2 mg; folic acid, 2 mg; nicotic acid, 60 mg; pantothenic acid, 18 mg; choline, 1800 mg. Added minerals and vitamins per kilogram of grower diet: Mn, 100 mg; I, 1 mg; Fe, 40 mg; Zn, 100 mg; Se, 0.15 mg; Cu, 10 mg; vitamin A, 12,000 IU; vitamin D_3_, 5000 UI; vitamin E, 50 mg; vitamin K_3_, 3 mg; vitamin B_1_, 2 mg; vitamin B_2_, 6 mg; vitamin B_6_, 4 mg; vitamin B_12_, 0.016 mg; biotin, 0.2 mg; folic acid, 1.75 mg; nicotic acid, 60 mg; pantothenic acid, 18 mg; choline, 1600 mg. Added minerals and vitamins per kilogram of finisher diet: Mn, 100 mg; I, 1 mg; Fe, 40 mg; Zn, 100 mg; Se, 0.15 mg; Cu, 10 mg; vitamin A ,12,000 IU; vitamin D_3_, 5000 UI; vitamin E, 50 mg; vitamin K_3_, 2 mg; vitamin B_1_, 2 mg; vitamin B_2_, 5 mg; vitamin B_6_, 3 mg; vitamin B_12_, 0.011 mg; biotin, 0.05 mg; folic acid, 1.5 mg; nicotic acid, 35 mg; pantothenic acid, 18 mg; choline, 1600 mg

^e^ME_n_ = metabolizable energy (ME) in the diets corrected to zero nitrogen balance

^f^Analyzed values. Each value based on triplicate determinations

One female broiler chicken and one male broiler chicken with the body weight close to the average were selected from each cage for the dissection, which was carried out according to the method described by Ziołecki and Doruchowski [13]. For slaughter analysis, the breast and drumstick muscles and liver were sampled and the basic nutrients and selected mineral elements were determined.

### Tissue Sampling Procedure and Chemical Analyses

The tissue samples of the breast muscle (m. pectoralis major and m. pectoralis minor), drumstick muscle (m. gastrocnemius, m. peroneus longus, and m. tibialis anterior), and liver were harvested immediately after slaughter, frozen at a temperature of −18 °C, and stored until chemical analyses. The muscles used in the analyses were without skin with the subcutaneous fat tissue. Three replicates of each sample were analyzed and the mean value was used in the data analyses. The contents of dry matter, total protein, ether extract, and crude ash were determined in the muscles and liver according to standard procedures [14]. The content of nutrients in the samples was expressed in grams per 100 g wet tissue. The energy value was estimated using net Atwater equivalents (considering protein and fat).

The contents of Ca, Mg, Fe, Zn, and Cu in the tissue and diets were measured (three replicates of each sample) using flame atomic absorption spectrophotometry (FAAS) (Unicam 939/959AA-6300, Shimadzu Corp., Tokyo, Japan), according to the Polish Norm PN-EN ISO 6869. Tissue samples, dried at 65 °C for the following 24 h and then at 105 °C for 24 h, underwent combined mineralization in a muffle furnace at a temperature of 450 °C for 12 h, using hydrogen peroxide as an oxidant. The resulting ash was dissolved in 1 M HNO_3_. Calcium was determined at *λ* = 422.7 nm, magnesium at *λ* = 285.2 nm, iron at *λ* = 248.3 nm, zinc at *λ* = 213.9 nm, and copper at *λ* = 324.8 nm [15]. The method accuracy was evaluated using minerals determined in the standard reference material chicken meat NCS ZC73016. Total P content was determined colorimetrically PN-76/R-64781 [16] with a Helios Alpha UV-vis apparatus (Spectronic Unicam, Leeds, UK). The content of macro- and microminerals in the samples was expressed in milligrams per 100 g wet tissue.

### Statistical Analysis

The data obtained were elaborated with the ANOVA method using one-way analysis of variance (*α* = 95; *P* < 0.05) and calculating the mean values for the treatments ($\overline{x}$) and the standard error of the mean (SEM). Linear and quadratic polynomial contrasts were used to evaluate the effects of different dietary levels of *B*. *serrata* resin. The direction and intensity of the relationships between the level of *B*. *serrata* resin addition and the basic nutrients and mineral elements (*r*_1_) in analyzed tissues and between each basic nutrient and mineral content in the broiler chicken meat and liver were determined using Pearson correlation coefficients (*r*_2_). The significance of differences was determined with Statistica 10.0 software StatSoft Inc. [17].

## Results

The range of the content of the basic nutrients determined in the breast and drumstick muscles and liver was typical for these tissues (Tables 2, 3, and 4). Similar concentrations of the basic nutrients, macroelements, and trace elements in muscles of broiler chickens were reported in the US National Nutrient Database for Standard Reference for raw chicken meat [18].

**Table 2:** Content of basic nutrients and mineral elements in the breast muscle of broiler chickens

Item	Treatment^a^				Statistical parameters			
	C	BSR1.5	BSR2	BSR2.5	SEM	*P* value C vs. BSR	Linear	Quadratic
Basic nutrients (g 100 g^−1^)								
Dry matter	25.89	26.08	26.17	26.18	0.67	0.123	0.578	0.674
Crude protein	23.72	23.46	23.15	23.09	0.61	0.264	0.315	0.418
Ether extract	1.03	1.07	1.11	0.95	0.03	0.037	0.293	0.175
Crude ash	1.12	1.09	1.14	1.13	0.07	0.319	0.214	0.378
Energy (kcal 100 g^−1^)	104.23	103.47	102.59	100.91	11.64	0.139	0.178	0.568
Mineral elements (mg 100 g^−1^)								
Ca	28.02	29.21	31.14	30.65	0.45	0.016	0.023	0.516
P	240.01	241.12	238.43	237.25	21.16	0.264	0.119	0.367
Mg	16.43	16.24	16.34	16.17	0.06	0.128	0.248	0.438
Fe	0.473	0.463	0.447	0.451	<0.01	0.091	0.078	0.248
Zn	0.496	0.506	0.513	0.521	<0.01	0.148	0.487	0.569
Cu	0.045	0.041	0.034	0.033	<0.01	0.033	0.024	0.196

Data represent the mean of ten broiler chickens per treatment

^a^Treatments: C = control diet without *Boswellia serrata* supplementation; BSR1.5 = diet with 1.5% *B*. *serrata* supplementation; BSR2 = diet with 2% *B*. *serrata* supplementation; BSR2.5 = diet with 2.5% *B*. *serrata* supplementation

**Table 3:** Content of basic nutrients and mineral elements in the drumstick muscle of broiler chickens

Item	Treatment^a^				Statistical parameters			
	C	BSR1.5	BSR2	BSR2.5	SEM	*P* value C vs. BSR	Linear	Quadratic
Basic nutrients (g 100 g^−1^)								
Dry matter	26.04	25.54	26.04	26.23	0.38	0.164	0.297	0.458
Crude protein	19.32	18.84	18.91	18.23	0.57	0.109	0.187	0.238
Ether extract	5.69	5.63	5.15	4.84	0.04	0.026	0.048	0.267
Crude ash	1.03	1.01	0.98	0.96	0.06	0.192	0.248	0.549
Energy (kcal 100 g^−1^)	128.49	126.03	121.95	116.48	12.34	0.035	0.036	0.248
Mineral elements (mg 100 g^−1^)								
Ca	8.07	8.25	8.76	8.39	0.33	0.041	0.027	0.349
P	194.12	197.43	201.47	209.02	19.36	0.089	0.054	0.129
Mg	21.11	22.07	22.56	23.78	0.04	0.024	0.019	0.467
Fe	0.647	0.649	0.648	0.653	0.02	0.139	0.346	0.247
Zn	1.520	1.507	1.479	1.434	0.05	0.027	0.029	0.178
Cu	0.078	0.073	0.062	0.051	<0.01	0.016	0.018	0.331

Data represent the mean of ten broiler chickens per treatment

^a^Treatments: C = control diet without *Boswellia serrata* supplementation; BSR1.5 = diet with 1.5% *B*. *serrata* supplementation; BSR2 = diet with 2% *B*. *serrata* supplementation; BSR2.5 = diet with 2.5% *B*. *serrata* supplementation

**Table 4:** Content of basic nutrients and mineral elements in the liver of broiler chickens

Item	Treatment^a^				Statistical parameters			
	C	BSR1.5	BSR2	BSR2.5	SEM	*P* value C vs. BSR	Linear	Quadratic
Basic nutrients (g 100 g^−1^)								
Dry matter	23.36	23.43	23.70	24.71	0.43	0.098	0.079	0.273
Crude protein	19.12	19.22	19.33	20.16	0.31	0.167	0.487	0.349
Ether extract	2.99	3.05	3.08	3.13	0.02	0.234	0.189	0.647
Crude ash	1.23	1.22	1.21	1.23	0.09	0.109	0.378	0.458
Energy (kcal 100 g^−1^)	103.39	104.33	105.04	108.81	14.31	0.213	0.077	0.389
Mineral elements (mg 100 g^−1^)								
Ca	23.45	23.16	24.23	25.89	0.52	0.044	0.187	0.026
P	317.23	324.61	334.72	341.50	26.48	0.067	0.093	0.647
Mg	19.47	19.78	20.34	21.63	0. 46	0.037	0.034	0.288
Fe	4.63	4.59	4.53	4.48	0.21	0.119	0.458	0.394
Zn	3.87	3.91	3.96	4.09	0.16	0.256	0.349	0.261
Cu	0.67	0.69	0.53	0.49	0.02	0.023	0.189	0.039

Data represent the mean of ten broiler chickens per treatment

^a^Treatments: C = control diet without *Boswellia serrata* supplementation; BSR1.5 = diet with 1.5% *B*. *serrata* supplementation; BSR2 = diet with 2% *B*. *serrata* supplementation; BSR2.5 = diet with 2.5% *B*. *serrata* supplementation

*B*. *serrata* resin (BSR2.5) supplementation at the level of 2.5% in broiler chicken diets decreased the ether extract in breast muscles (control vs. BSR diets, linear, *P* < 0.05) (Table 2). The other nutrients determined in the muscles of chickens from all the treatment groups exhibited a similar level. Although there were no statistically significant differences in the energy value of the breast muscles of chickens supplemented with BSR, there was a strong negative correlation between the calorific value and the level of BSR supplementation (*r*_1_ = −0.686). There was an effect of the *B*. *serrata* resin on the Ca (control vs. BSR diets, linear, *P* < 0.05) and Cu (control vs. BSR diets, linear, *P* < 0.05) contents in breast muscles (Table 2). Additionally, a strong correlation was found between the content of these mineral elements in the breast muscle and the BSR level in the chicken diets (*r*_1_ = 0.865 and *r*_1_ = −0.841, respectively). The P, Mg, Fe, and Zn contents in the breast muscles of the broiler chickens remained at a similar level, regardless of the application and the amount of the supplement. Simultaneously, the Zn content was positively correlated with the level of BRS supplementation (*r*_1_ = 0.586).

As in the case of the breast muscles, the addition of *B*. *serrata* resin to the broiler chicken diets decreased the ether extract in the drumstick muscles (control vs. BSR diets, linear, *P* < 0.05) (Table 3). These changes were reflected in the calorific value of the drumstick muscles (control vs. BSR diets, linear, *P* < 0.05). The increasing proportion of BSR in the broiler chicken diets had a beneficial effect on the dietary parameters of the drumstick muscles, which was confirmed by the estimated correlation coefficients: *r*_1_ = −0.945 (ether extract) and *r*_1_ = 0.971 (energy). The drumstick muscles of broiler chickens fed with BSR supplementation were characterized by a higher level of Ca and Mg (control vs. BSR diets, linear, *P* < 0.05) and lower contents of Zn and Cu (control vs. BSR diets, linear, *P* < 0.05), compared with the muscles of the broiler chickens in the C treatments. Additionally, the amount of Zn and Cu microelements in the drumstick muscles was accumulated in inverse proportion to the BSR level in the chicken diet (*r*_1_ = −0.745 and *r*_1_ = −0.830, respectively).

No changes in the nutrient content were noted in the analyzed livers (Table 4). The BSR supplementation altered liver Ca, Mg, and Cu accumulations in the broilers at 42 days of age (*P* < 0.05). Compared with the control treatment, the inclusion of BSR significantly increased Ca and Mg accumulation in the liver (respectively, control vs. BSR diets, quadratic, *P* < 0.05; *r*_1_ = 0.713 and control vs. BSR diets, linear, *P* < 0.05; *r*_1_ = 0.521). In turn, the content of Cu decreased in the liver with the increasing BSR level (control vs. BSR diets, quadratic, *P* < 0.05; *r*_1_ = −0.776). The BSR treatments did not have a significant effect on retention of the other micro- and macroelements determined.

The values of the correlation coefficients (*r*_2_) between basic nutrients and some elements in the breast and drumstick muscles and liver of chicken broilers supplemented with BSR are presented in Tables 5 and 6. Strong correlations were found between the content of the individual basic nutrients and elements in the muscles. A high negative correlation (*r*_2_ > −0.7) was observed in the case of the following pairs: Ca-Ca, Zn; P-Mg, Fe, crude ash; Cu-Mg; Fe-Mg, P, Fe, crude ash; Zn-dry matter; dry matter-Mg, Fe, crude ash; crude protein-crude protein, crude ash; ether extract-Mg, P, Fe, crude protein, crude ash; and crude ash-Cu, dry (breast-drumstick muscles, respectively; *P* < 0.05) (Table 5). Strong positive correlations (*r*_2_ > 0.7) were noted for Ca-Mg, Cu, Fe, crude protein; Mg-Ca, Zn; P-Zn; Cu-dry matter, ether extract; Fe-Ca, Cu, ether extract; dry matter-Zn; and ether extract-Ca, Zn, ether extract (breast-drumstick muscles, respectively; *P* < 0.05).

**Table 5:** Correlation coefficients between basic nutrients and mineral elements in the breast and drumstick muscles of BSR-supplemented broiler chickens (*r*_2_), significance values *P* < 0.05

		Breast muscles									
		Ca	Mg	P	Cu	Fe	Zn	Dry matter	Crude protein	Ether extract	Crude ash
Drumstick muscles	Ca	−0.901	0.788	ns	ns	0.762	ns	ns	0.675	0.963	ns
	Mg	0.929	−0.687	−0.771	−0.767	−0.767	ns	−0.952	−0.670	−0.915	ns
	P	ns	−0.628	−0.699	ns	−0.799	ns	ns	ns	−0.893	ns
	Cu	0.731	ns	ns	ns	0.733	ns	ns	ns	0.697	−0.778
	Fe	0.830	−0.645	−0.769	ns	−0.866	ns	−0.870	ns	−0.896	ns
	Zn	−0.776	0.896	0.725	ns	ns	−0.677	0.801	ns	0.799	ns
	Dry matter	ns	ns	ns	0.858	ns	−0.824	ns	ns	ns	−0.843
	Crude protein	0.812	−0.798	ns	ns	−0.610	ns	ns	−0.814	−0.886	ns
	Ether extract	−0.659	ns	ns	0.706	0.954	ns	ns	ns	0.741	ns
	Crude ash	0.664	ns	−0.811	ns	−0.807	ns	−0.818	−0.620	−0.839	ns

*ns* no statistical significance

**Table 6:** Correlation coefficients between basic nutrients and mineral elements in the liver and the breast and drumstick muscles of BSR-supplemented broiler chickens (*r*_2_), significance values *P* < 0.05

		Liver									
		Ca	Mg	P	Cu	Fe	Zn	Dry matter	Crude protein	Ether extract	Crude ash
Breast muscles	Ca	ns	ns	ns	ns	ns	ns	ns	0.684	0.613	ns
	Mg	−0.897	ns	ns	ns	ns	ns	−0.646	−0.715	ns	ns
	P	−0.777	−0.793	−0.899	0.782	0.824	−0.896	−0.670	ns	−0.781	−0.760
	Cu	−0.754	−0.806	−0.857	0.897	0.737	−0.790	ns	ns	−0.851	ns
	Fe	−0.778	ns	−0.702	0.786	0.826	−0.697	−0.648	−0.756	−0.782	ns
	Zn	0.876	ns	0.846	−0.972	−0.799	0.940	0.714	ns	0.672	ns
	Dry matter	ns	ns	ns	ns	ns	ns	ns	−0.948	ns	ns
	Crude protein	ns	ns	ns	ns	ns	ns	ns	ns	ns	ns
	Ether extract	−0.801	−0.638	ns	ns	0.683	−0.870	−0.603	−0.761	−0.812	ns
	Crude ash	0.669	ns	0.825	−0.929	ns	ns	ns	ns	0.655	ns
Drumstick muscles	Ca	−0.611	−0.686	ns	−0.611	ns	ns	−0.754	−0.813	−0.626	ns
	Mg	0.617	0.692	ns	ns	ns	ns	0.759	0.817	0.632	ns
	P	0.785	0.897	0.616	−0.807	−0.839	0.699	0.813	ns	0.788	0.733
	Cu	−0.788	−0.667	−0.897	0.755	0.876	−0.960	ns	−0.613	ns	ns
	Fe	0.743	0.806	ns	ns	−0.614	ns	0.860	0.604	0.782	0.682
	Zn	−0.822	−0.874	−0.665	ns	0.709	−0.887	−0.718	−0.651	−0.850	−0.650
	Dry matter	−0.680	−0.604	ns	0.734	0.798	0.798	ns	ns	ns	ns
	Crude protein	ns	0.520	ns	ns	ns	ns	ns	0.674	ns	ns
	Ether extract	−0.696	−0.981	ns	0.635	0.897	−0.675	ns	ns	−0.794	ns
	Crude ash	0.667	0.738	ns	ns	ns	0.756	0.800	0.631	0.681	ns

*ns* no statistical significance value

Strong inversely proportional correlations (*r*_2_ > −0.7) were determined in the case of Ca-Mg, P, Cu, Fe, ether extract; Mg-P, Cu; P-P, Cu, Fe; Cu-Zn, crude ash; Fe-Zn; Zn-P, Cu, ether extract; crude protein-Mg, Fe, dry matter, ether extract; ether extract-P, Cu, Fe, ether extract; and crude ash-P between the liver and breast muscles (*P* < 0.05) and between the liver and drumstick muscles: Ca-Cu, Zn; Mg-Zn, ether extract; P-Cu; Cu-P; Fe-P; dry matter-Ca, Zn; crude protein-Ca; and ether extract-Zn, ether extract (*P* < 0.05) (Table 6). In turn, there were high positive correlations (*r*_2_ > 0.7) between the contents of mineral elements and nutrients in the liver and breast muscles: Ca-Zn; P-Zn, crude ash; Cu-P, Cu, Fe; Fe-P, Cu, Fe; Zn-Zn; and dry matter-Zn (*P* < 0.05) and in the liver and drumstick muscles: Ca-P, Fe; Mg-P, Fe, crude ash; Cu-Cu, dry matter, ether extract; Fe-Cu, Zn, dry matter; Zn-dry matter, crude ash; dry matter-Mg, P, Fe, crude ash; crude protein-Mg; and ether extract-P, Fe and crude ash-P (*P* < 0.05).

## Discussion

Higher dietary values of the poultry meat resulting from the BSR supplementation of broiler chicken diets were noted in all the analyzed tissues, i.e., both in the meat and liver. The decreased content of crude fat in the broiler chicken tissues and, hence, its reduced calorific value may be associated with the anti-inflammatory and bacteriostatic properties of the most active components, i.e., boswellic acids [19, 20]. Although there are no investigations concerning the potential application of BSR in animal production, laboratory analyses prove that *B*. *serrata* resin and its extracts can have a significant effect on lipid metabolism in animals. Tests carried out on rats have revealed a positive impact of the use of this type of supplementation, i.e., reduction of the serum cholesterol and triglyceride levels [21]. Additionally, researchers have shown that *B*. *serrata* can stimulate the function of the thyroid, leading to metabolic upregulation and increased calorie burning. The beneficial effect of *B*. *serrata* on the cholesterol and triglyceride levels is also related to improvement in liver function, which can result in reduction of cholesterol biosynthesis and stimulation of transformation of cholesterol into bile acids. Although the exact mechanisms of action have not been elucidated yet, these results prove the positive impact of *B*. *serrata* on animal health and the quality of food products of animal origin. *B*. *serrata* is beginning to arouse researchers’ interest due to its potential therapeutic and dietary applications in humans, e.g., in the treatment of diabetes or even obesity [22–24].

The improvement in the dietary value of the breast and drumstick muscle and liver can also be associated with the antioxidant and immunomodulatory activity of boswellic acid, which has a beneficial impact on stabilization of the gastrointestinal microbial flora [25]. In their previous investigations, the authors observed a positive effect of BSR on the structure of intestinal villi, gastrointestinal microbiome, and health status in broiler chickens, which was reflected in higher feed nutrient intake and greater rearing performance [9, 10]. There are no scientific reports on the effect of the BSR active compounds on mineral metabolism in animals; yet, investigations on the application of various phytobiotics (herbs, essential oils, and oleoresins) in poultry nutrition confirm the influence of these additives on the level of element retention in tissues and their status in the organism. The mechanisms of phytobiotics include alteration in the gastrointestinal functions, induction and inhibition of metabolic enzymes, beneficial modification in the intestinal microbiota, increased digestibility and nutrient absorption, histological modifications of the gastrointestinal tract, and even stimulation of appetite [9, 10, 26–29].

The presence of BSR in broiler chicken diets, in particular at the levels of 2 and 2.5%, increased the Ca and P contents in the muscle tissues of the broiler chickens. An optimal level of these elements in the organism determines, e.g., a normal course of digestive processes and maintenance of appropriate homeostasis in the broiler organism [30]. The high Ca content in the broiler chicken meat accompanied by reduced fat content may be nutritionally advantageous. The analyzed tissues, i.e., the breast and drumstick muscles and liver, exhibited negative correlations between the Ca content and the ether extract (*r*_2_ > 0.6). Calcium compounds and fat in the gastrointestinal tract can form insoluble products that block Ca availability on the one hand and impair the digestive availability of fat on the other hand, which can be taken advantage of in body-weight reduction diets [31, 32]. Increased Mg levels were noted in the drumstick muscles of the BSR-supplemented broiler chickens. This element is mainly accumulated in bones; in tissues, Mg is involved in neuronal and cardiac muscle excitability, release of hormones and neurotransmitters, and normal muscle contractility, as it is responsible, together with Ca, for normal muscle function. In birds, Mg deficiency leads to muscle weakness, tremors, or even spasms, which indicates health problems in the flock and results in reduced productivity [33, 34]. A high correlation between the contents of these elements was found in the case of the analyzed breast and drumstick muscles, which seems to be advantageous for breeding performance (Ca-Mg: *r*_2_ = 0.929 and Mg-Ca: *r*_2_ = 0.788). The increased levels of these elements in the muscles of the broiler chickens may have resulted in improved overall health status and production performance, which was reported by the authors in their previous study [10].

All the tissues were characterized by a reduced level of the analyzed microelements: Cu (muscles and liver), Fe (breast muscle and liver), and Zn (drumstick muscle). Besides their specific biochemical role, the elements cooperate to protect cells against excess amounts of free oxygen radicals, regulate the activity of cellular enzymes, and reinforce the hematopoietic role of Fe [35, 36]. However, despite the reduction of their level in the tissues, no health deterioration was observed in birds studied previously. On the contrary, beneficial effects of BSR supplementation on weight gain and dressing percentage or better feed conversion rates were noted [10]. As indicated in the literature, these effects are also observed in the case of reduced bioavailability of these elements [37–39]. This prompts new areas of research aimed at investigating whether BSR supplementation can contribute to enhancement of mineral bioavailability in feed. Mohite et al. [40] and Surai [41] suggest that enhanced bioavailability of minerals may also result from reduction in the concentration of polyphenols, flavonoids, and tannins in the gastrointestinal tract associated with microbial fermentation. The effect of phytobiotics on mineral metabolism in the broiler chicken organism can be multidirectional. Similar to the present study, reduction of Fe levels in chicken breast muscles caused by supplementation with aqueous herbal extracts (Galega [Herb. Galegae], stinging nettle [Herb. Utricae], lemon balm [Fol. Melissae], and sage [Fol. Salviae]) was reported by Wójcik et al. [42]. Similar results were reported by Starčević et al. [43], who supplemented broiler chicken diets with active compounds isolated from phytobiotics, i.e., thymol, tannic acid, and gallic acid. In turn, Giannenas et al. [44], who used oregano essential oil and laurel essential oil as feed additives, did not observe changes in the content of Cu and Zn in breast and thigh meat. The differences in the impact of phytobiotics on retention of trace elements in animal muscles are associated with the high diversity of the phytobiotic active compounds, i.e., terpenoids (mono- and sesquiterpenes, steroids, etc.), alkaloids (alcohols, aldehydes, ketones, esters, ethers, lactones, etc.), glycosides, and phenolics (tannins). They can exert different effects on the availability of mineral components [26, 27, 29, 45, 46].

Interactions in the content of nutrients in the animal organisms depend on the species, breed, sex, rearing system, health status, and the physiological-biochemical function of muscles and organs [47–49]. The interrelations between nutrients in the organism are strongly influenced by their content and availability in the diet as well as the status of the gastrointestinal microbiome, which can be optimized by application of appropriate dietary additives [5, 10, 50, 51]. In the investigations conducted by Herkel et al. [5], significant correlations were found between dry matter with crude protein and fat in breast and thigh muscles of fattening turkeys fed with a diet supplemented with a blend of essential oils from origanum, anise, and citrus fruits and fructooligosaccharides. The present study did not reveal such an effect of *B*. *serrata* resin on the interactions between nutrients in the muscles. Herkel et al. [5] also recorded positive correlations between ash and phosphorus, ash and copper, and ash and iron (*P* < 0.01). Such significant correlations between ash and minerals in the breast and drumstick muscles (crude ash-Cu; P-crude ash; Fe-crude ash) were found in this study, but they were reverse. Among the determined nutrients, significant correlations were most frequently found between the mineral elements and ether extract. They were usually negative and were noted mainly for Mg, P, and Fe. The results may suggest that the accumulation of mineral elements in the muscles and liver was higher at the lower fat content, which can undoubtedly increase the nutritional values of meat. However, further research in this area is indispensable to confirm these correlations. High correlations were noted between the levels of mineral elements in the breast and drumstick muscles; they were especially strong in the case of the Ca, Mg, P, and Fe contents. Połtowicz and Doktor [52] report that Mg in the poultry muscle tissue regulates the level of intracellular Ca and P; they noted especially strong Mg-P correlations. These correlations had a significant effect on the meat processing quality, as they regulated the water retention capacity of the analyzed meat. A strong correlation between Fe-Cu, Cu-Cu, and Cu-Zn in the breast and drumstick muscles of chickens fed with diets supplemented with various phytobiotics was reported by Stef et al. [27]. These results, however, confirm the great diversity of mechanisms regulating retention of mineral elements in the organism of broiler chickens, as the BSR supplementation analyzed in the present study did not cause such correlations. The strong correlations between basic nutrient and mineral elements in the breast and drumstick muscles observed in the present study may be an effect of the improvement of digestibility stimulated by the addition of BSR in the diets [10]. This effect may differentiate the chemical composition of leg and breast muscles. Greater intensity of these changes was noted for the leg muscles. Chicken breast and leg muscles differ substantially in their histochemical profile and the character of metabolic processes. Similar differences in the levels of minerals in breast and leg muscles were reported by Zapata et al. [48] and Goluch et al. [53]. In the present study, there were clear correlations with the same direction between the components of the analyzed muscles (Ca-Mg, Mg-Ca, crude protein-Ca, crude protein-Mg, Ca-crude protein, and Mg-crude protein), which may have been related to their important role in enzymatic processes and their impact on the muscle tissue pH and protein hydration. Ca and Mg contribute to maintenance of osmotic pressure and electrolyte balance in cells and tissues; thus, they play a vital role in regulation of the meat hydration level [54]. There were strong correlations between the microelement content (in particular Cu and Fe) in the liver and in the breast and drumstick muscles in the broiler chickens fed with the BSR additive. The liver is characterized by substantially higher accumulation of microelements, especially Fe and Cu, than that in other organs. A similar phenomenon was also observed by Stef et al. [27], who found significant correlations between the content of Fe-Zn, Fe-Cu, Zn-Fe, and Cu-Fe in the liver of broiler chickens, regardless of the type of diet. Such correlations between microelements are not confined to poultry; they have also been found in the case of the liver of wild-living animals, i.e., Ca-Fe (−) and Zn-Cu (−) in boars; Ca-Cu (−), Ca-Fe (−), and Cu-Fe (+) in roe deer; and Ca-Cu (−), Zn-Cu, and Fe (+) in hares [55].

The calculated correlation coefficients indicate relationships whose strength and direction are related to the physicochemical properties of the elements as well as antagonistic or synergistic effects, bioavailability, co-occurrence, and involvement with other components in the physiological and metabolic processes in the animal organism.

## Conclusions

The use of the 2 and 2.5% addition of the *B*. *serrata* resin in the diets decreased the ether extract in the breast and drumstick muscles, as well as their calorific value. The BSR contributed to the increase in the Ca content in the analyzed tissues and the Mg content in the drumstick muscles and liver but decreased the muscular Cu and Zn (in drumstick muscles) retention. Although the present results confirm the beneficial effect of *B*. *serrata* resin on the dietary and nutritional value of poultry meat, they can be a starting point for further investigations aimed at elucidation of the mechanisms of the impact of the phytobiotic on the broiler chicken organism.
